# Supplementary material for: Structural insights into sequence-dependent Holliday junction resolution by the chloroplast resolvase MOC1
Source: Nat Commun. 2020 Mar 17;11:1417. doi: 10.1038/s41467-020-15242-8 (PMC7078210; doi:10.1038/s41467-020-15242-8)
Supplement: Supplementary file 1 — Supplementary Information [file 41467_2020_15242_MOESM1_ESM.pdf]

Supplementary Information

Structural insights into sequence-dependent Holliday junction resolution by the chloroplast resolvase MOC1

J. Yan, S. Hong, *et al.*,

Supplementary Figure 1

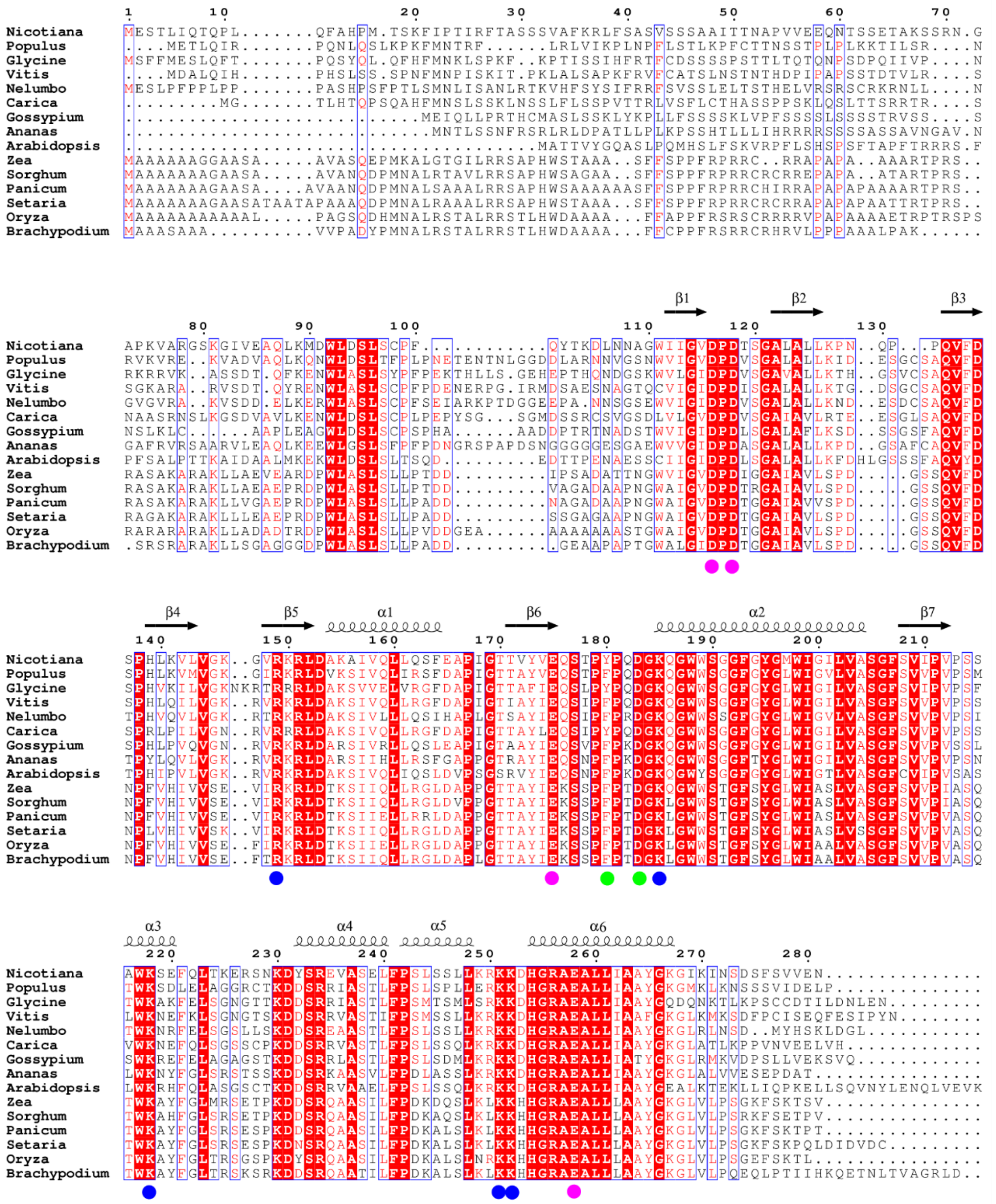

Supplementary Figure 1. Multiple sequence alignment of MOC1 homologs in plants. The sequences used for alignment are from the NCBI database. *Nicotiana tabacum* (XP_016490038.1), *Populus trichocarpa* (XP_002314007.2), Glycine max (XP_003532877.2), *Vitis vinifera* (XP_002263460.1), *Nelumbo nucifera* (XP_010271822.1), *Carica papaya* (XP_021900948.1), *Gossypium raimondii* (XP_012444423.1), *Ananas comosus* (XP_020102424.1), *Arabidopsis thaliana* (AT2G26840.1), *Zea mays* (ONM31293.1), *Sorghum bicolor* (OQU86680.1), *Panicum*

hallii (PAN31407.1), *Setaria italic* (XP_004967727.1), *Oryza sativa* (LOC_Os01g16340.1), *Brachypodium distachyon* (XP_003566861.1). The numbers above the sequence indicate the residue numbers of NtMOC1.The secondary structural elements are labeled above the sequence. The completely conserved amino acids are shaded in red. Magenta circles indicate the catalytic tetrad. Green circles indicate residues involved in sequence-specific cleavage of HJ. Blue circles indicate the basic residues involved in HJ binding and catalysis. Sequence alignment was performed by MultAlin.

Supplementary Figure 2

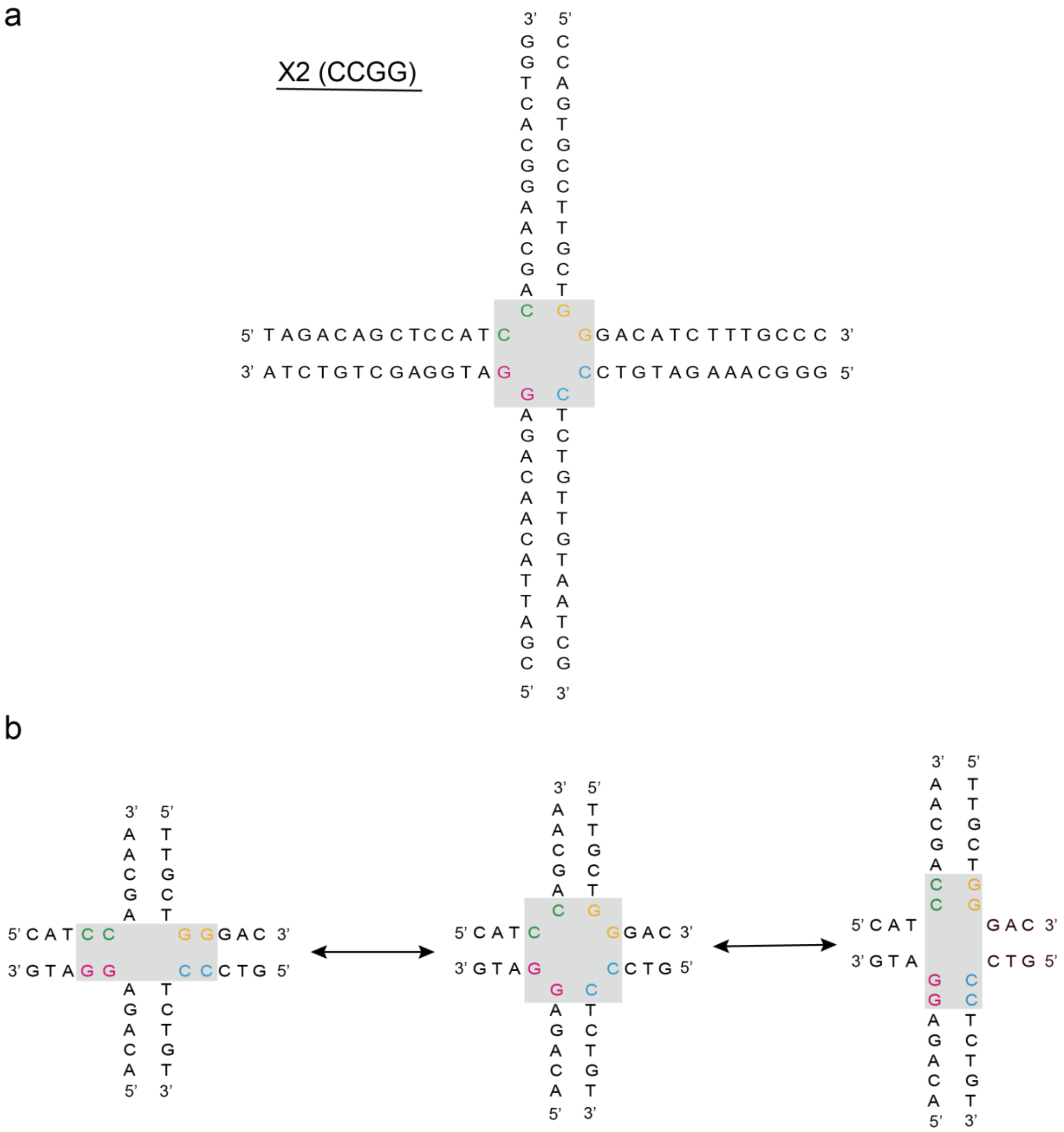

Supplementary Figure 2. Bimobility of the X2 HJ substrates. a, Schematic drawing of the X2 (CCGG) substrate. X2 HJ was prepared by annealing 4 oligos with 2-bp homologous core sequence. **b**, Branch migration of the X2 (CCGG). X2 harbors 2-bp homologous core sequence (gray background) that exhibits branch migration of two steps within the core sequence. Other X2 substrates used in this study exhibit identical bimobile characteristics as the X2 (CCGG).

Supplementary Figure 3

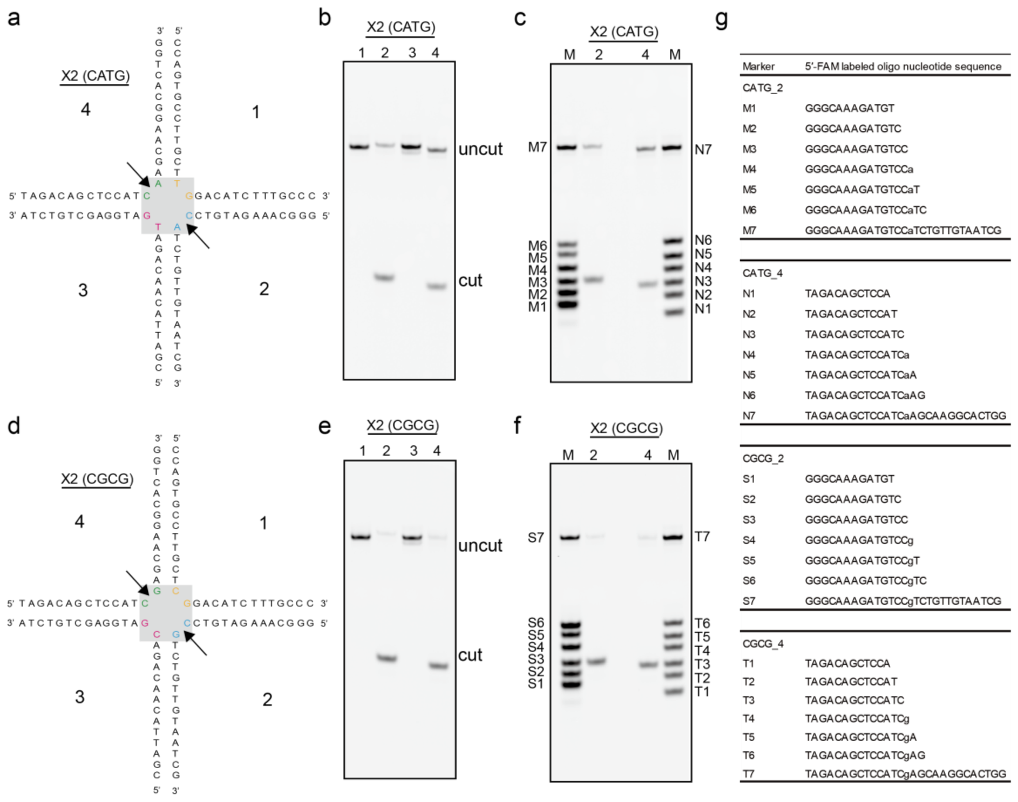

Supplementary Figure 3. Cleavage site mapping for junction X2 (CATG) and X2 (CGCG). a,d, Schematic drawing of the X2 (CATG) and X2 (CGCG) substrates. Arrows indicate the cleavage site revealed by the following mapping assays. **b,e,** Strands 2 and 4 of the X2 (CATG) and X2 (CGCG) are cleavable DNA strands. The 5′ end of one of the DNA strands (1, 2, 3 or 4) was labeled with FAM. The concentration of HJ in each reaction is 250 nM. Protein concentration is 1000 nM. AtMOC1 was used as a representative for the mapping assay. The reaction products were resolved by denaturing PAGE. **c,f,** Cleavage site mapping for strands 2 and 4 of the X2 (CATG) and X2 (CGCG). The reaction products were resolved by denaturing PAGE and mapped by 5′-FAM labeled oligo sequences. **g,** 5′-FAM labeled oligo nucleotide sequences used as marker for cleavage site mapping. Source data are provided as a Source Data file.

Supplementary Figure 4

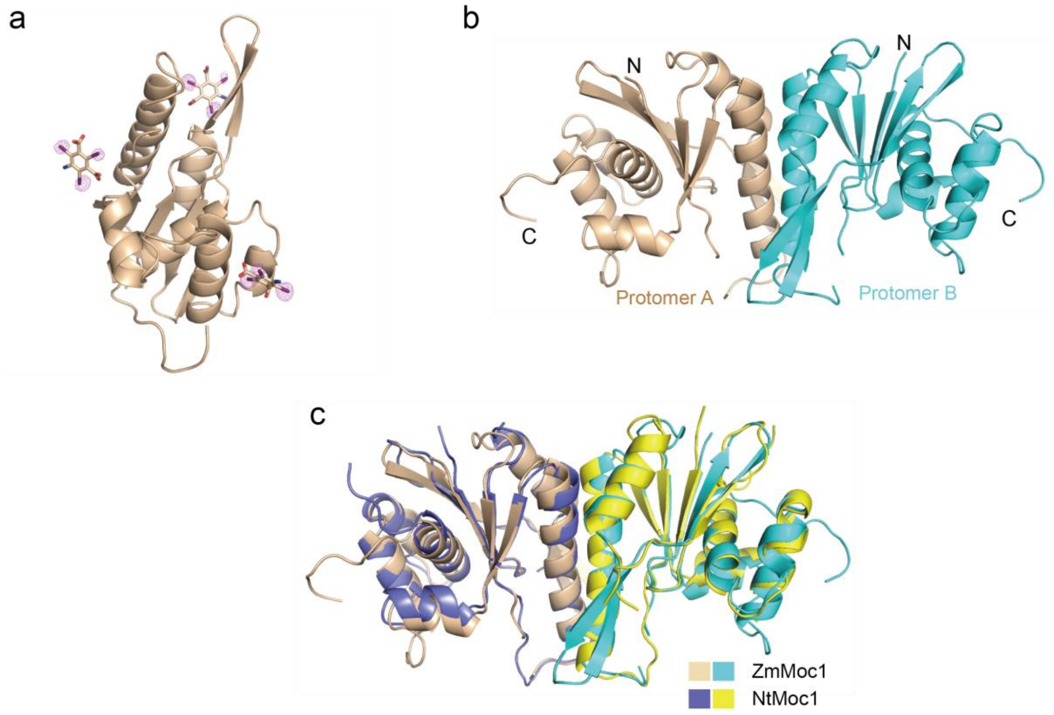

Supplementary Figure 4. Crystal structure of ZmMOC1. a, Electron density map of I3C compounds in ZmMOC1. The map is contoured at 3σ (pink). The equilateral triangle formed by the I atom is clearly visible. **b,** Crystal structure of ZmMOC1 (T107-V280) determined in this study. Protomer A and B are colored in wheat and cyan, respectively. The same color scheme is used for the following panel of ZmMOC1. **c,** Structural superimposition of NtMOC1 with ZmMOC1. Protomer A and B of NtMOC1 are colored in slate and yellow, respectively. Structural alignment reveals an RMSD value of 0.919 Å over 257 Cα.

Supplementary Figure 5

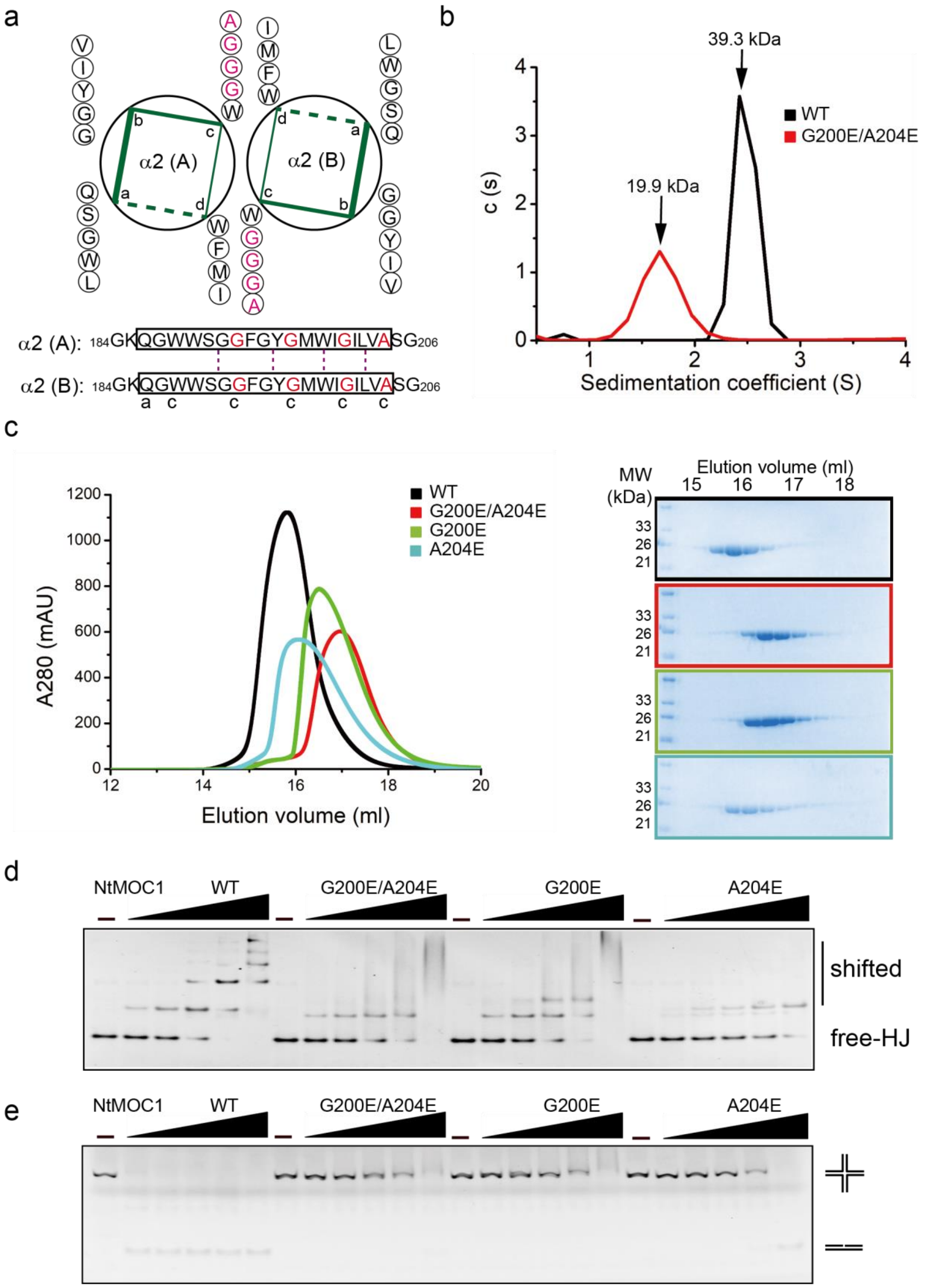

Supplementary Figure 5. Disruption of the NtMOC1 dimerization affects its HJ resolution activity. a, Helical wheel presentation of the helix bundle from α2 of both molecules. Black and orange indicate the hydrophobic and uncharged residues. Amino acids sequences are shown below

and numbered. The hydrophobic interactions between the residues of α2 from both protomers are linked with dashed lines. **b,** The molecular mass of wild-type and mutant NtMOC1 examined by AUC experiments. The theoretical molecular weight of a monomeric NtMOC1 (V76-N281) is 22.5 kDa; c(s) represents the continuous (sedimentation coefficient distribution) analysis model. **c,** SEC analysis of the dimeric states for the mutant NtMOC1. Fractions at the same elution volume from individual injections were examined by SDS–PAGE. **d,** HJ-binding activity of the mutants with impaired dimerization. **e,** HJ cleavage activity of the mutants with impaired dimerization. For the HJ binding and cleavage assay, X2 (CCGG) was used as the HJ substrate. The final concentration of HJ in each lane is 250 nM. Six gradients with increasing concentrations (0, 500, 1000, 2000, 4000, and 8000 nM) were applied for each protein sample. The reactions were resolved by native PAGE and visualized by GelRed staining. Source data are provided as a Source Data file.

Supplementary Figure 6

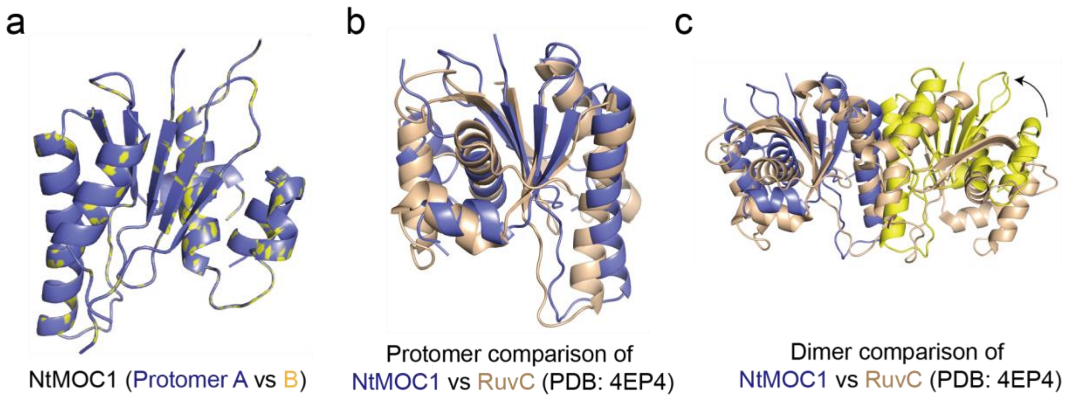

Supplementary Figure 6. Structural alignment. a, Structural superimposition of protomer A and B of apo NtMOC1. **b,** Structural superimposition of the protomers between NtMOC1 and RuvC. NtMOC1 and RuvC are colored slate and brown, respectively. **c,** Structural superimposition of the dimeric NtMOC1 with dimeric RuvC. NtMOC1 is shown in slate (protomer A) and yellow (protomer B). RuvC is shown in brown.

Supplementary Figure 7

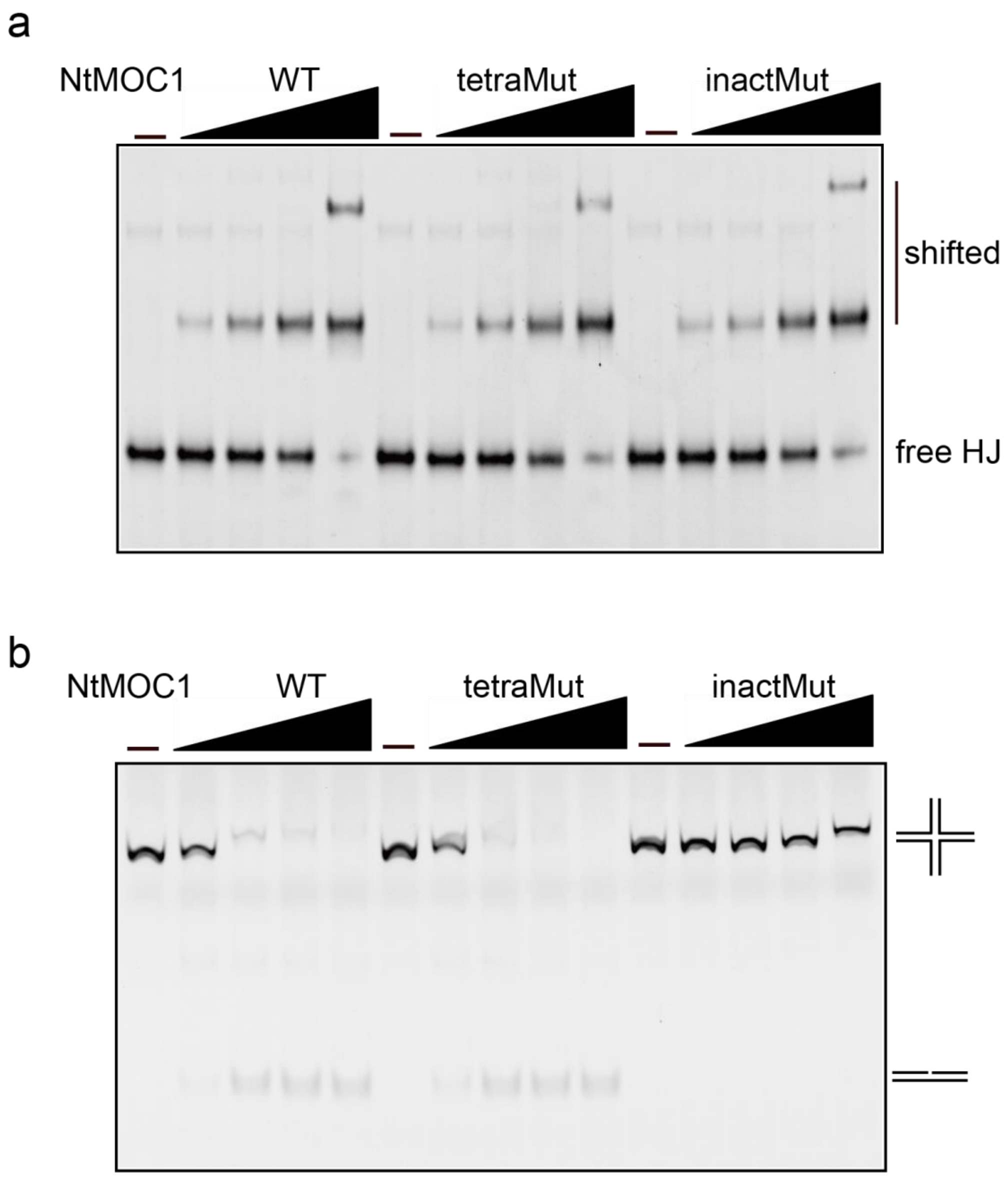

Supplementary Figure 7. HJ binding and cleavage activity of NtMOC1 mutants used for co-crystallization with HJs. a, HJ binding activity of NtMOC1 mutants. **b,** HJ cleavage activity of NtMOC1 mutants. WT, wild-type NtMOC1. tetraMut, NtMOC1 (I112V; Q162K; E235Q; 239Q). inactMut, NtMOC1 (I112V; Q162K; E235Q; 239Q; D116A; E175A; D253A; E258A). For the HJ binding and cleavage assay, X2 (CCGG) was used as the HJ substrate. The final concentration of HJ in each lane is 250 nM. Five gradients with increasing concentrations (0, 125, 250, 500, and 1000 nM) were applied for each protein sample. The reactions were resolved by native PAGE and visualized by GelRed staining. Source data are provided as a Source Data file.

Supplementary Figure 8

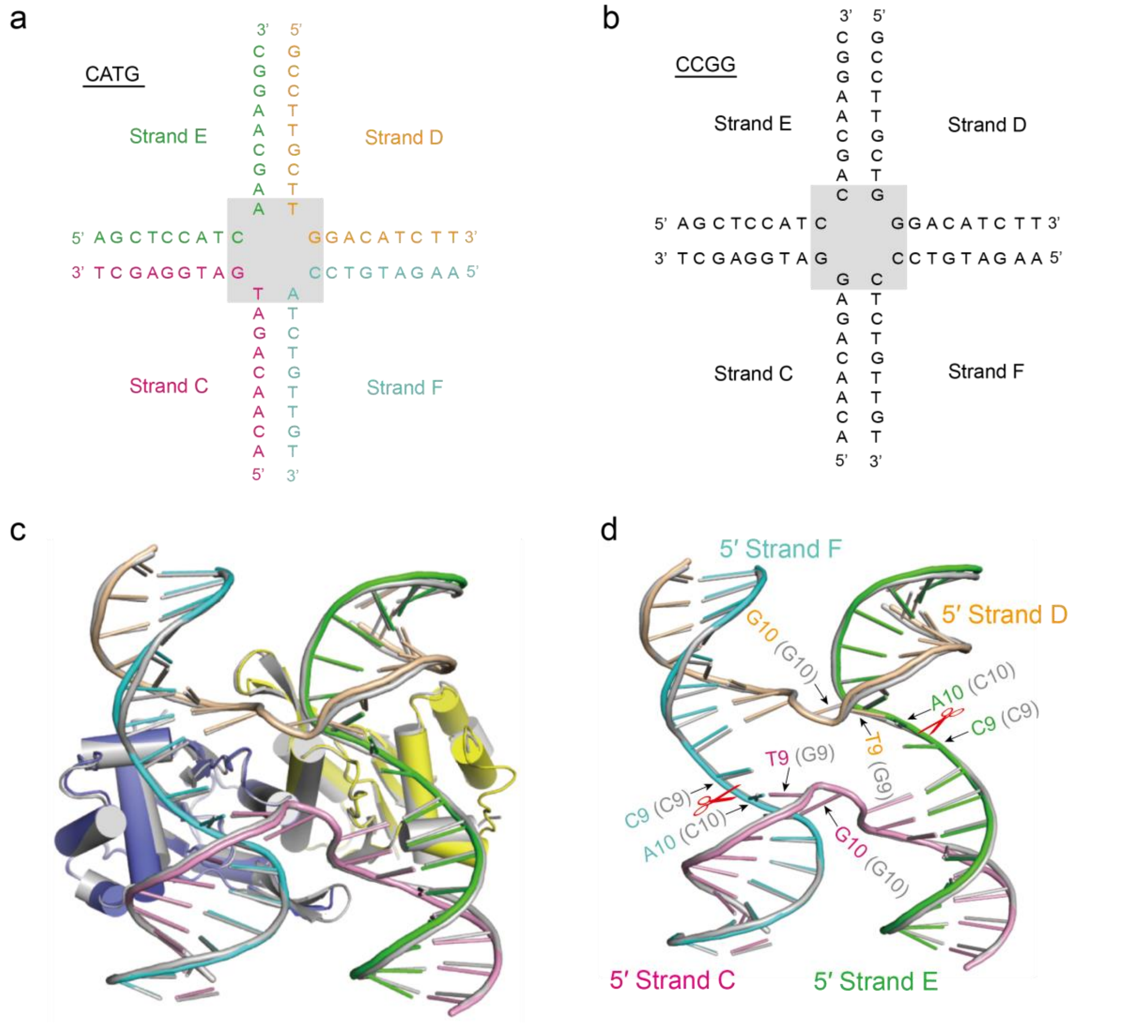

Supplementary Figure 8. Structure comparison of NtMOC1 in complex with HJs with cognate sequence or non-cognate sequence. a,b, Schematic drawing of HJs crystallized in the complex structure with either non-cognate CATG core sequence (**a**) or cognate CCGG core sequence (**b**). The 2 bp homologous core sequences are highlighted in gray box. **c,** Structure superimposition of NtMOC1-HJ complexes. The colored cartoon indicates the complex structure of NtMOC1 and HJ with non-cognate CATG core sequence. The gray cartoon indicates the complex structure of the cleavage inactive NtMOC1 mutant (D116A/E175A/D253A/E258A) and HJ with cognate CCGG core sequence. **d,** Structure alignment of HJs with CATG and CCGG cores. The colored cartoon indicates the HJ with CATG core, whereas gray cartoon indicates the HJ with CCGG core. The CATG and CCGG cores at the branch point are labeled, and the nucleotides can be well aligned. Scissors indicate the cleavage site.

Supplementary Figure 9

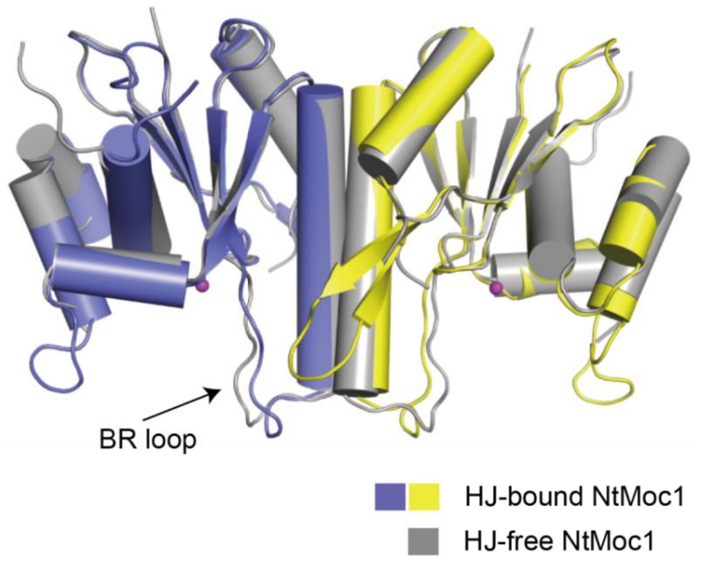

Supplementary Figure 9. Structural superimposition of the apo and HJ-bound NtMOC1. HJ-bound NtMOC1 is shown in slate (protomer A) and yellow (protomer B). The apo NtMOC1 is colored gray.

Supplementary Figure 10

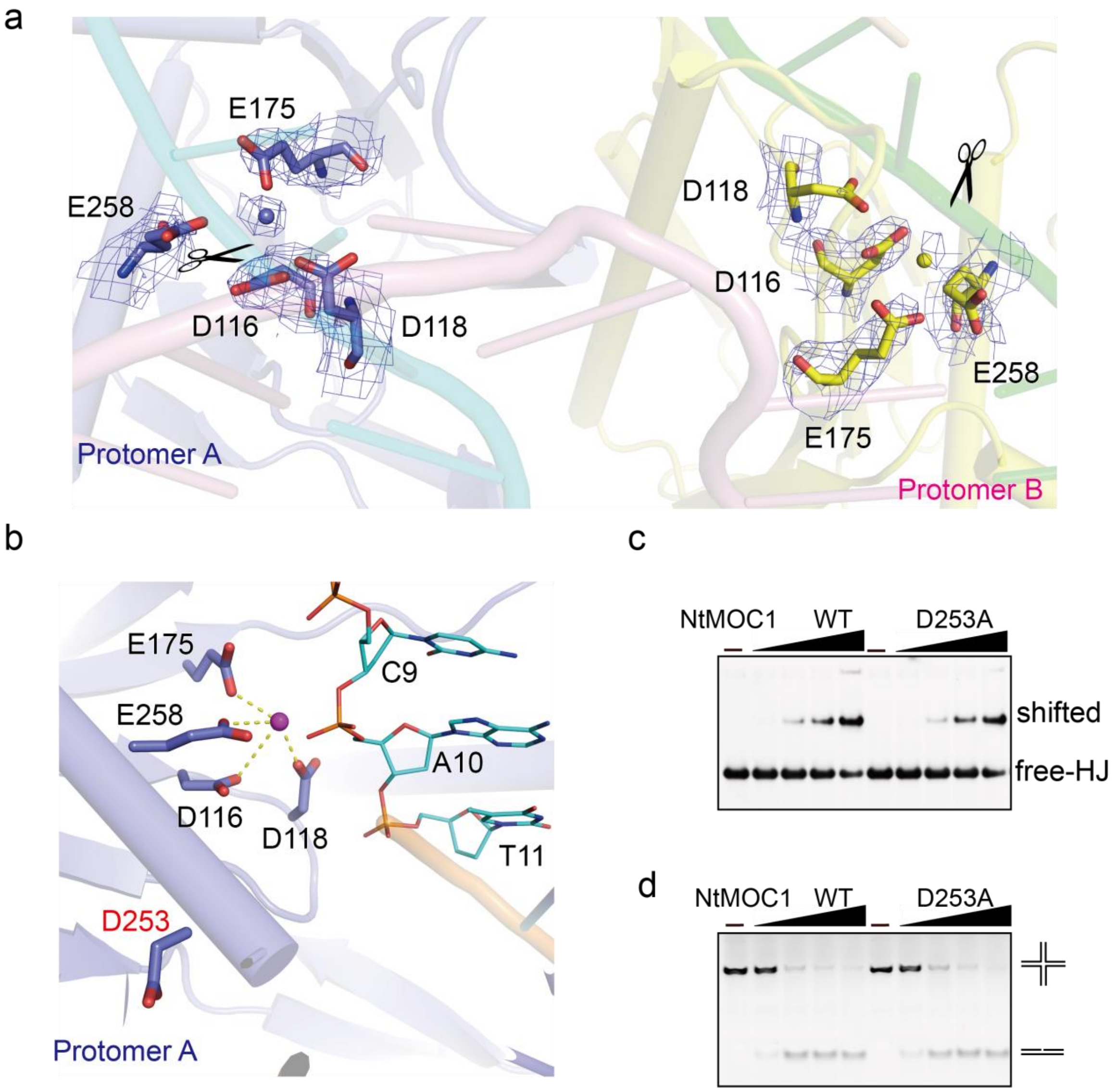

Supplementary Figure 10. Catalytic center of NtMOC1. a, The electron density of the catalytic tetrad and the magnesium ion. The map is contoured at 1.0 σ. The density of the magnesium ion in protomer A is much clearer than that in protomer B. Scissors indicate the cleavage site. **b,** D253 is neighboring the active site. **c,d,** Mutant of D253A exhibit no effects on HJ binding (**c**) or resolution activity (**d**). For the HJ binding and cleavage assay, X2 (CCGG) was used as the HJ substrate. The final concentration of HJ in each lane is 250 nM. Five gradients with increasing concentrations (0, 125, 250, 500, and 1000 nM) were applied for each protein sample. The reactions were resolved by native PAGE and visualized by GelRed staining. Source data are provided as a Source Data file.

Supplementary Figure 11

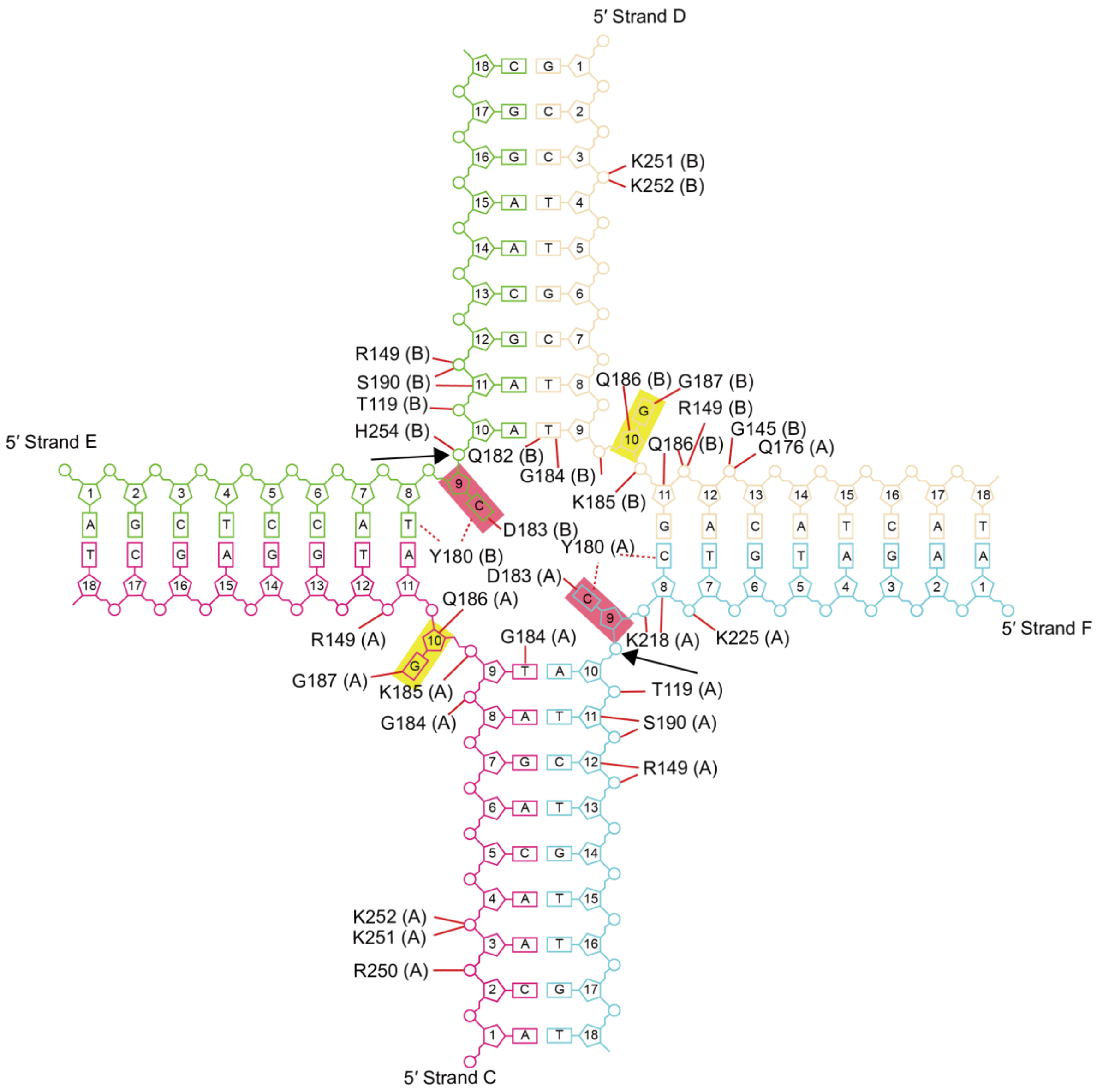

Supplementary Figure 11. Schematic representation of protein-DNA interactions in the NtMOC1-HJ complex. The DNA sequence of each strand is shown, and the bases are sequentially labeled in the ribose rings from the 5′ side. Residues involved in contact with the DNA backbone and bases are labeled. The solid and dashed lines indicate hydrogen bonds and base stacking interactions, respectively. Arrows indicate the cleavage site. The C-G base pairs at the crossover are disrupted. The determinant cytosine recognized by D183 is shaded in magenta. The out flipped guanine is shaded in yellow.

Supplementary Figure 12

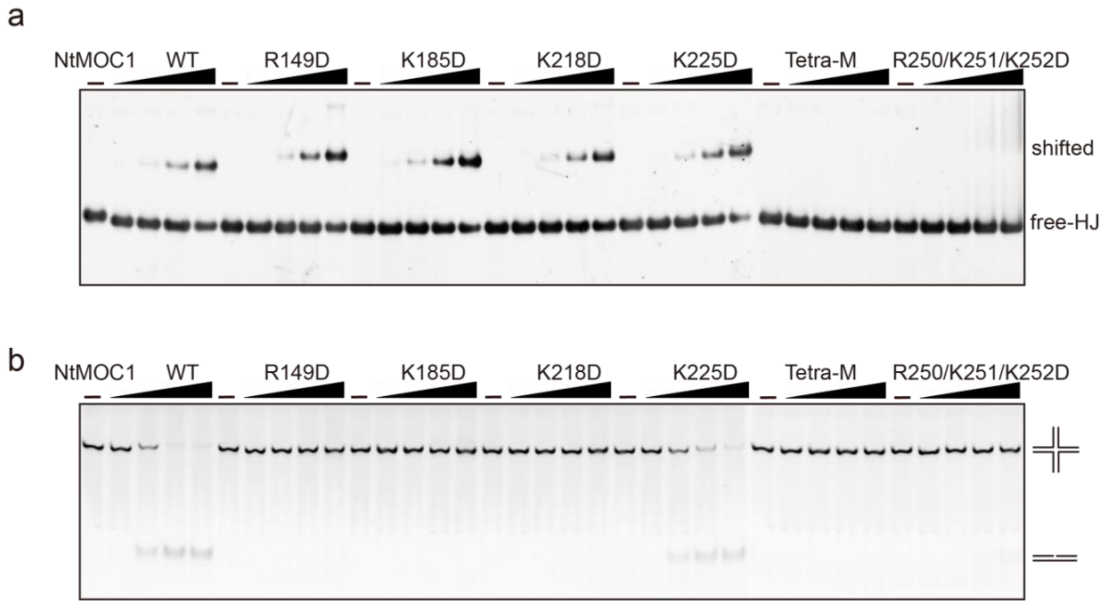

Supplementary Figure 12. Charge-reversal mutations at the surface of NtMOC1 affect the HJ-binding and resolution activity. a, HJ binding activity. **b,** HJ resolution activity. Relative mutations are labeled above. Tetra-M indicates a tetra-mutation (R149D/K185D/K218D/K225D). For the HJ binding and cleavage assay, X2 (CCGG) was used as the HJ substrate. The final concentration of HJ in each lane is 250 nM. Five gradients with increasing concentrationS (0, 125, 250, 500, and 1000 nM) were applied for each protein sample. The reactions were resolved by native PAGE and visualized by GelRed staining. Source data are provided as a Source Data file.

Supplementary Figure 13

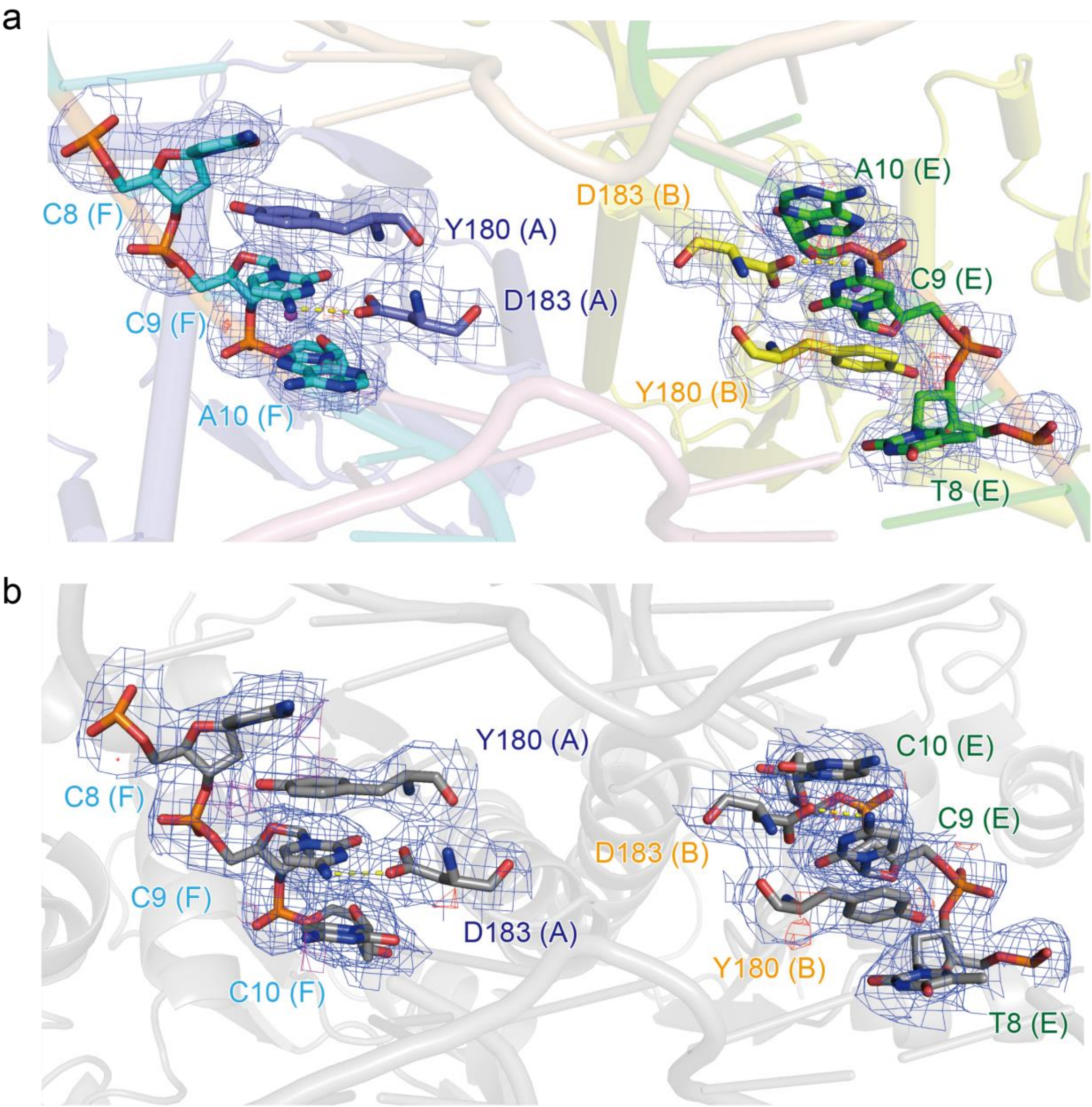

Supplementary Figure 13. Electron density map of base recognition residues and the bases at the crossover of the NtMOC1-HJ complexes. a, The superimposed 2Fo-Fc (1.0 σ, lightblue) and Fo-Fc (±3.0 σ, purple and red) map of key elements from the complex structure composed of non-cognate CATG core sequence. **b,** The superimposed 2Fo-Fc (1.0 σ, lightblue) and Fo-Fc (±3.0 σ, purple and red) map of key elements from the complex structure composed of cognate CCGG core sequence. Residues and bases are labeled.

Supplementary Figure 14

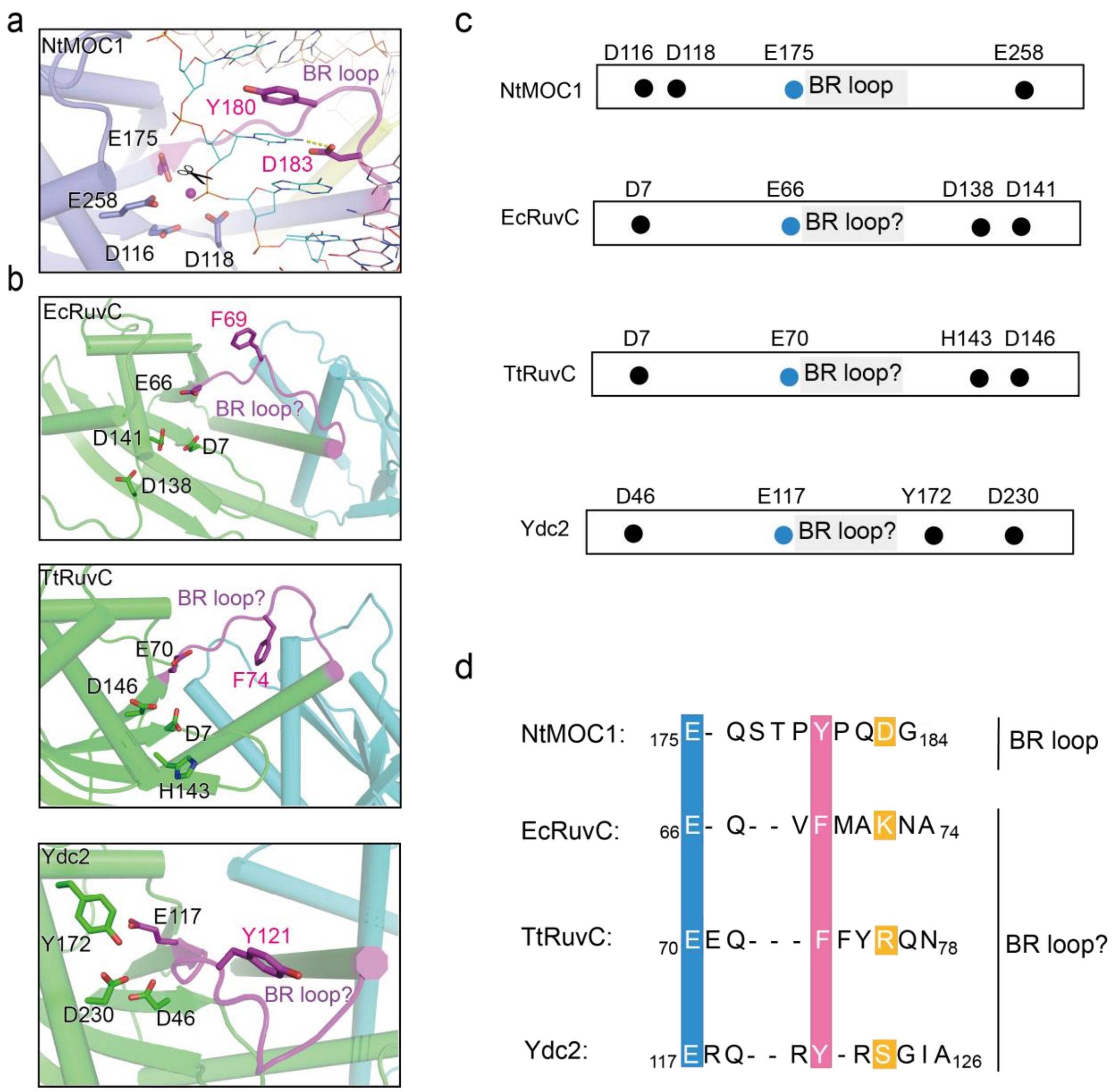

Supplementary Figure 14. Similar BR loops are present in other members of retroviral integrase superfamily resolvase. a, Structural features of the BR loop in NtMOC1. Residues constituting the catalytic tetrad are shown in black. The BR loop is colored in purple. Residues Y180 and D183 from the BR loop, protruding into the junction and forming stacking and hydrogen bond interactions with the bases, are colored in magenta. **b,** Structural features of a similar loop in EcRuvC (PDB ID: 1hjr), TtRuvC (PDB ID: 4ep4), and Ydc2 (PDB ID: 1kcf). Residues constituting the catalytic tetrad are shown in black. The potential BR loop is colored in purple. An aromatic residue (Tyr/Phe) from the loop potentially forming base stacking interactions is shown in magenta. **c,** The schematic diagram of the resolvases NtMOC1, EcRuvC,

TtRuvC, and Ydc2. The catalytic residues are shown with circles and labeled above. BR loop following the catalytic glutamate is shaded in light gray. **d,** Sequence alignment of the BR loop. The catalytic glutamate is shaded blue, whereas the aromatic and charged/polar residues potentially involved in base stacking and base recognition are shaded in magenta and orange, respectively. Amino acids numbers are labeled.

Supplementary Figure 15

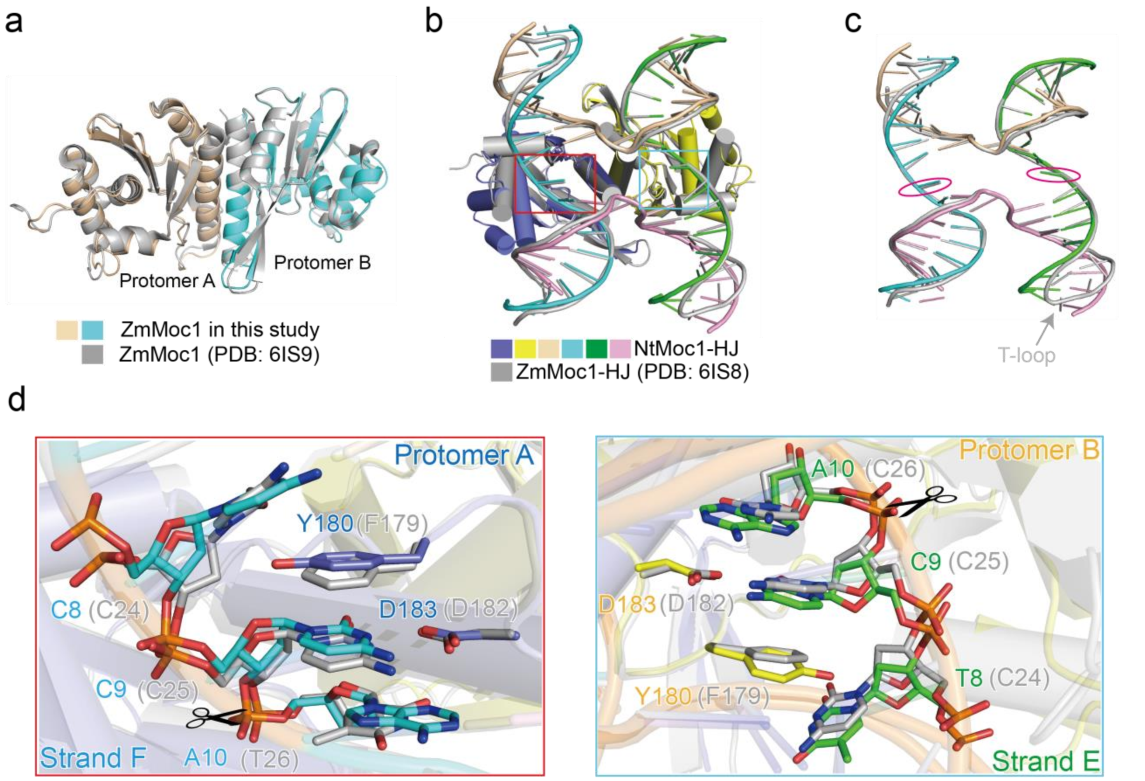

Supplementary Figure 15. Structural comparisons of NtMOC1-HJ and ZmMOC1-HJ complex. a, Structural comparison of iodide-phased ZmMOC1 (by our study) with selenium-phased ZmMOC1 (PDB ID: 6IS9). For clarity, residues 86-95 from protomer A of 6IS9 are omitted. **b,** Structural superimposition of the complex structure of NtMOC1-HJ and ZmMOC1-HJ. **c,** Structural alignment of four oligos annealed HJ with two oligos annealed (with a T-loop) HJ in the complex structures. The magenta oval highlights the disrupted cytosine at the crossover of both HJs. The arrow indicates a T-loop. **d,** Structural alignment of the crucial residues and the bases involved in the sequence-specific HJ resolution. The colored cartoon represents the structure of NtMOC1-HJ determined in this study. The gray cartoon represents the structure of ZmMOC1-HJ determined by Lin group (PDB ID: 6IS8). Scissors indicate the scissile phosphates.

Supplementary Table 1 Data collection, refinement and structural determination

	ZmMOC1 (6LCM)	NtMOC1 (6KVN)	NtMOC1 - HJ^{catg} (6KVO)	$NtMOC1_{inact}$ - HJ^{ccgg} (6LCT)[#]
**Data collection**				
Space group	$P4_32_12$	$p2_12_12$	$p2_12_12$	$p2_12_12$
Cell dimensions				
a, *b*, *c* (Å)	49.14, 49.14, 141.83	60.50, 62.79, 40.33	121.32, 65.89, 92.15	121.5, 89.81, 68.48
α, β, γ (°)	90.00, 90.00, 90.00	90.00, 90.00, 90.00	90.00, 90.00, 90.00	90.0, 90.00, 90.00
Resolution (Å)	45-2.5 (2.59-2.5)	45~2.02 (2.07~2.02)	45~2.5 (2.6~2.5)	45~2.55 (2.66~2.55)
Rmerge(%)	9.2 (30.2)	19.2 (80.9)	23.0 (46.3)	3.3 (72.7)
I/σ (*I*)	70.8 (19)	6.0 (2.6)	5.3 (2.2)	28.2 (2.6)
Completeness (%)	100 (100)	99.8 (98.3)	99.8 (98.9)	99.9 (99.9)
Redundancy	33.8 (13.5)	6.3 (5.7)	6.5 (6.6)	6.5 (6.7)
**Refinement**				
Resolution (Å)	45-2.5	45~2.02	45~2.5	45~2.55
No. measured reflections	221911 (22800)	66548 (4218)	171614 (19235)	175,724 (19,934)
No. unique reflections	6,571 (1689)	10538 (737)	26297 (2898)	26865 (2958)
Rwork/Rfree (%)	22.27/27.99	19.15/23.67	20.19/23.43	21.99/24.49
No. atoms				
Protein	1264	1154	2492	2365
DNA	0	0	1468	1465
Water	53	61	47	9
B-factors				
Protein	26.8	42.0	85.6	104.8
DNA	0	0	102.4	121.3
Water	29.5	47.7	80.0	90.0
R.m.s. deviations				
Bond lengths (Å)	0.006	0.007	0.008	0.003
Bond angles (°)	0.684	0.774	0.940	0.517

Values in parentheses are for the highest resolution shell.

[#]inact, inactive, represent the NtMOC1 mutant with inactive cleavage activity.

Supplementary Table 2 Homologous structure search for NtMOC1 by Dali

No.	Chain	Z-score	rmsd	lali	nres	%id	Description
1	4ktw-A	16.2	2.7	147	160	15	RUVC ENDONUCLEASE;
2	4ep4-A	13.6	3	143	166	14	ENDODEOXYRIBONUCLEASE RUVC;
3	5e6f-A	12.4	2.7	122	130	12	CNPV261 HOLLIDAY JUNCTION RESOLVASE PROTEIN;
4	5ola-A	11.3	3.2	143	212	16	TRANSCRIPTION ELONGATION FACTOR;
5	1kcf-A	10.5	3.4	140	240	11	HYPOTHETICAL 30.2 KD PROTEIN C25G10.02 IN
6	3bzk-A	9.8	3	114	728	11	TEX;
7	1ng9-A	9.2	2.6	107	794	11	5'-D(*AP*GP*CP*TP*GP*CP*CP*AP*GP*GP*CP*AP*CP*CP*A
8	1vhx-A	8.3	4.6	123	140	11	PUTATIVE HOLLIDAY JUNCTION RESOLVASE;
9	2o8e-B	8.3	3.1	118	935	8	5'-D(*GP*AP*AP*CP*CP*GP*CP*GP*GP*GP*CP*TP*AP*GP*G
10	4oo8-D	8	4.1	137	1163	15	CRISPR-ASSOCIATED ENDONUCLEASE CAS9/CSN1;
11	6n9a-B	7.8	3.6	109	211	12	TRNA THREONYLCARBAMOYLADENOSINE BIOSYNTHESIS
12	5axw-A	7.8	4	134	1043	10	CRISPR-ASSOCIATED ENDONUCLEASE CAS9;
13	5x2g-A	7.7	3.5	115	749	14	CRISPR-ASSOCIATED ENDONUCLEASE CAS9;
14	3cpe-A	7.4	3	115	553	13	DNA PACKAGING PROTEIN GP17;
15	4czm-A	7.4	4.7	123	336	15	ROD SHAPE-DETERMINING PROTEIN MREB;
16	5oe9-C	7.3	4.5	118	415	10	LARGE SUBUNIT TERMINASE;
17	5b2o-A	7.2	4.2	128	1455	7	CRISPR-ASSOCIATED ENDONUCLEASE CAS9;
18	2wbn-A	7.1	3.2	111	178	11	TERMINASE LARGE SUBUNIT;
19	3zeu-A	7.1	3.5	109	230	9	PUTATIVE M22 PEPTIDASE YEAZ;
20	4m7x-A	7.1	3	98	273	8	TYPE II PANTOTHENATE KINASE;
21	6omv-B	7.1	3.5	126	1208	10	CRISPR-Cas12a (Cpf1)
22	4oge-A	7.1	3.6	127	977	11	HNH ENDONUCLEASE DOMAIN PROTEIN;

- Chain: structure and chain identifiers of the hits (matched proteins)
- Z-score: the summary list is sorted by Z-score. Z-scores above 7 are reported. Hits with higher Z-scores are more similar to the query.

- rmsd: root-mean-square-deviation of structurally equivalent C-alpha atoms in 3-D superimposition
- lali: number of structurally equivalent C-alpha atoms
- nres: number of residues in the target structure
- %id: percentage of identical amino acids out of structurally equivalent residues
- Description: echoed from the COMPND record of the PDB file

Supplementary Table 3 The nucleotide sequence used for HJ assembly in this study

(1) Oligos used for cleavage assays		
X2 (CCGG)	Sequence (5′- 3′)	Length (nt)
X2_CCGG_1	CCAGTGCCTTGCTGGGACATCTTTGCCC	28
X2_CCGG_2	GGGCAAAGATGTCCCTCTGTTGTAATCG	28
X2_CCGG_3	CGATTACAACAGAGGATGGAGCTGTCTA	28
X2_CCGG_4	TAGACAGCTCCATCCAGCAAGGCACTGG	28
X2 (CATG)		
X2_CATG_1	CCAGTGCCTTGCTTGGACATCTTTGCCC	28
X2_CATG_2	GGGCAAAGATGTCCATCTGTTGTAATCG	28
X2_CATG_3	CGATTACAACAGATGATGGAGCTGTCTA	28
X2_CATG_4	TAGACAGCTCCATCAAGCAAGGCACTGG	28
X2 (CGCG)		
X2_CGCG_1	CCAGTGCCTTGCTCGGACATCTTTGCCC	28
X2_CGCG_2	GGGCAAAGATGTCCGTCTGTTGTAATCG	28
X2_CGCG_3	CGATTACAACAGACGATGGAGCTGTCTA	28
X2_CGCG_4	TAGACAGCTCCATCGAGCAAGGCACTGG	28
X2 (CTAG)		
X2_CTAG_1	CCAGTGCCTTGCTAGGACATCTTTGCCC	28
X2_CTAG_2	GGGCAAAGATGTCCTTCTGTTGTAATCG	28
X2_CTAG_3	CGATTACAACAGAAGATGGAGCTGTCTA	28
X2_CTAG_4	TAGACAGCTCCATCTAGCAAGGCACTGG	28
X2 (ACGT)		
X2_ACGT_1	CCAGTGCCTTGCTGTGACATCTTTGCCC	28
X2_ACGT_2	GGGCAAAGATGTCACTCTGTTGTAATCG	28
X2_ACGT_3	CGATTACAACAGAGTATGGAGCTGTCTA	28
X2_ACGT_4	TAGACAGCTCCATACAGCAAGGCACTGG	28
X2 (GCGC)		
X2_GCGC_1	CCAGTGCCTTGCTGCGACATCTTTGCCC	28
X2_GCGC_2	GGGCAAAGATGTCGCTCTGTTGTAATCG	28
X2_GCGC_3	CGATTACAACAGAGCATGGAGCTGTCTA	28
X2_GCGC_4	TAGACAGCTCCATGCAGCAAGGCACTGG	28
X2 (TCGA)		
X2_TCGA_1	CCAGTGCCTTGCTGAGACATCTTTGCCC	28
X2_TCGA_2	GGGCAAAGATGTCTCTCTGTTGTAATCG	28
X2_TCGA_3	CGATTACAACAGAGAATGGAGCTGTCTA	28
X2_TCGA_4	TAGACAGCTCCATTCAGCAAGGCACTGG	28

(2) 5′ FAM labeled oligos for cleavage site mapping		
X2 (CATG)_FAM	5′ FAM labeled oligo sequence (5′- 3′)	
X2_CATG_1FAM	CCAGTGCCTTGCTTGGACATCTTTGCCC	28
X2_CATG_2 FAM	GGGCAAAGATGTCCATCTGTTGTAATCG	28
X2_CATG_3 FAM	CGATTACAACAGATGATGGAGCTGTCTA	28
X2_CATG_4 FAM	TAGACAGCTCCATCAAGCAAGGCACTGG	28
Markers for X2_CATG_2 cleavage site mapping		
M1	GGGCAAAGATGT	12
M2	GGGCAAAGATGTC	13
M3	GGGCAAAGATGTCC	14
M4	GGGCAAAGATGTCCa	15
M5	GGGCAAAGATGTCCaT	16
M6	GGGCAAAGATGTCCaTC	17
M7	GGGCAAAGATGTCCaTCTGTTGTAATCG	28
Markers for X2_CATG_4 cleavage site mapping		
N1	TAGACAGCTCCA	12
N2	TAGACAGCTCCAT	13
N3	TAGACAGCTCCATC	14
N4	TAGACAGCTCCATCa	15
N5	TAGACAGCTCCATCaA	16
N6	TAGACAGCTCCATCaAG	17
N7	TAGACAGCTCCATCaAGCAAGGCACTGG	28
X2 (CGCG)_FAM		
X2_CGCG_1FAM	CCAGTGCCTTGCTCGGACATCTTTGCCC	28
X2_CGCG_2FAM	GGGCAAAGATGTCCGTCTGTTGTAATCG	28
X2_CGCG_3FAM	CGATTACAACAGACGATGGAGCTGTCTA	28
X2_CGCG_4FAM	TAGACAGCTCCATCGAGCAAGGCACTGG	28
Markers for X2_CGCG_2 cleavage site mapping		
S1	GGGCAAAGATGT	12
S2	GGGCAAAGATGTC	13
S3	GGGCAAAGATGTCC	14
S4	GGGCAAAGATGTCCg	15
S5	GGGCAAAGATGTCCgT	16
S6	GGGCAAAGATGTCCgTC	17
S7	GGGCAAAGATGTCCgTCTGTTGTAATCG	28
Markers for X2_CGCG_4 cleavage site mapping		
T1	TAGACAGCTCCA	12
T2	TAGACAGCTCCAT	13
T3	TAGACAGCTCCATC	14
T4	TAGACAGCTCCATCg	15
T5	TAGACAGCTCCATCgA	16
T6	TAGACAGCTCCATCgAG	17
T7	TAGACAGCTCCATCgAGCAAGGCACTGG	28

(3) Oligos used for crystallization and structural determination		
HJ_CATG		
HJ_CATG_1	GCCTTGCTTGGACATCTT	18
HJ_CATG_2	AAGATGTCCATCTGTTGT	18
HJ_CATG_3	ACAACAGATGATGGAGCT	18
HJ_CATG_4	AGCTCCATCAAGCAAGGC	18
HJ_CCGG		
HJ_CCGG_1	GCCTTGCTGGGACATCTT	18
HJ_CCGG_2	AAGATGTCCCTCTGTTGT	18
HJ_CCGG_3	ACAACAGAGGATGGAGCT	18
HJ_CCGG_4	AGCTCCATCCAGCAAGGC	18
